# Supplementary material for: Carbon Nanodisks Decorated with Guanidinylated Hyperbranched Polyethyleneimine Derivatives as Efficient Antibacterial Agents
Source: Nanomaterials (Basel). 2024 Apr 13;14(8):677. doi: 10.3390/nano14080677 (PMC11054016; doi:10.3390/nano14080677)
Supplement: Supplementary file 1 [file nanomaterials-14-00677-s001.zip › nanomaterials-2950286-supplementary.pdf]

Carbon nanodisks decorated with guanidinylated hyperbranched polyethyleneimine derivatives as efficient antibacterial agents

Kyriaki-Marina Lyra [1], Ioannis Tournis[1], Mohammed Subrati[1], Konstantinos Spyrou [2], Aggeliki Papavasiliou[1], Chrysoula P. Athanasekou[1], Sergios Papageorgiou[1], Elias Sakellis[1,3] Michael A. Karakassides[2] and Zili Sideratou [1*]

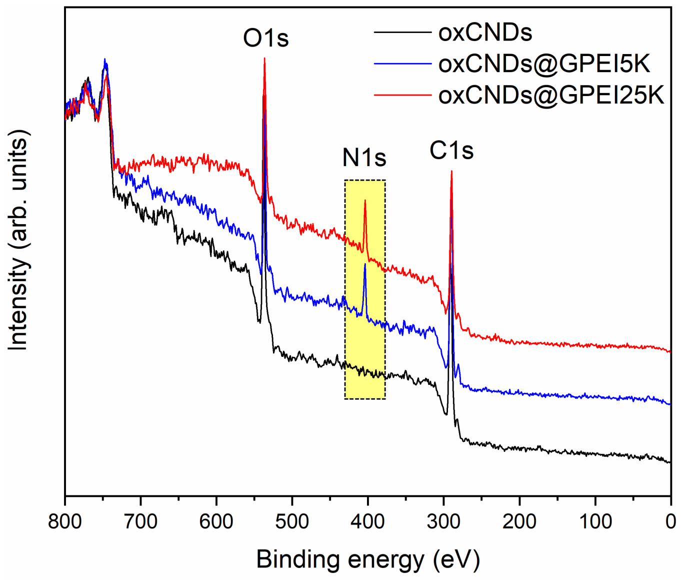

Figure S1. XPS survey spectra of oxCNDs, oxCNDs@GPEI5K, and oxCNDs@GPEI25K.

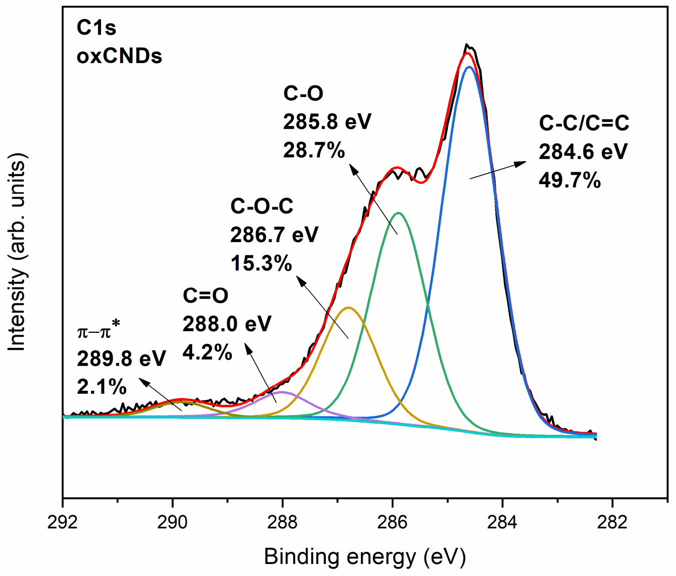

Figure S2. Deconvoluted high-resolution core-level C1s spectrum of oxCNDs.

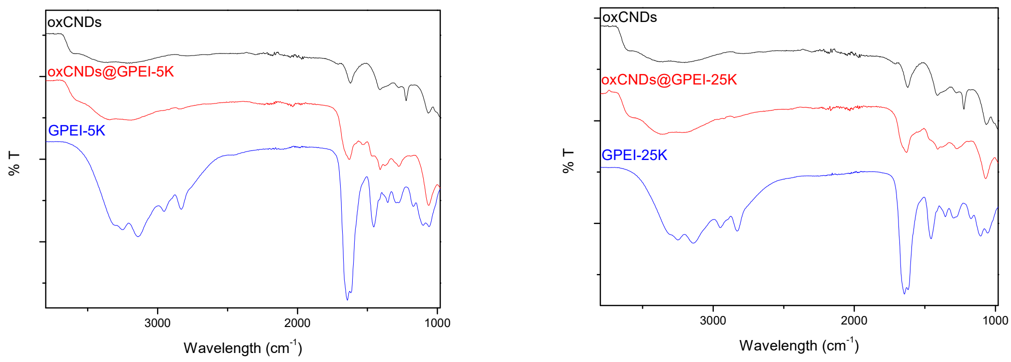

(**a**) (**b**)

Figure S3. FTIR spectra of (**a**) oxCNDs, GPEI5K and oxCNDs@GPEI5K and (**b**) oxCNDs, GPEI25K and oxCNDs@GPEI25K.

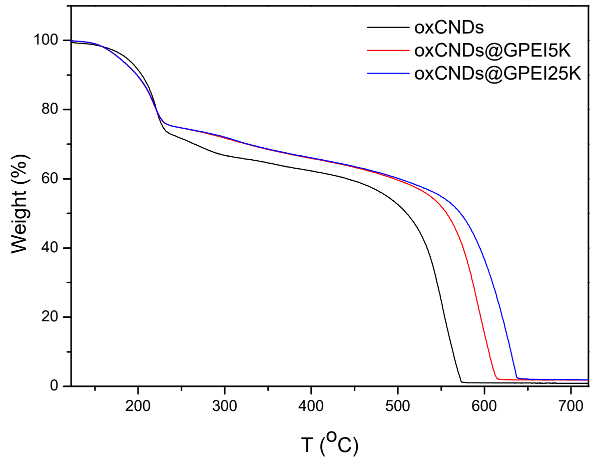

Figure S4. Thermogravimetric analysis (TGA) curves of oxCNDs, GPEIs and GPEI-functionalized oxCNDs.

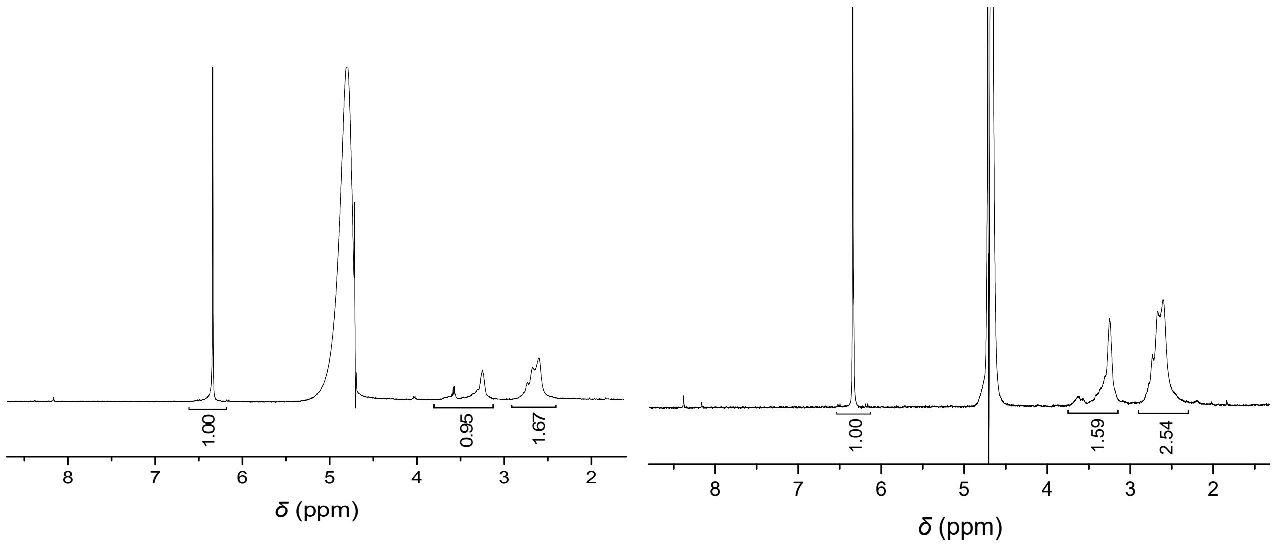

(**a**) (**b**)

Figure S5. ^{1}H NMR spectra (500 MHz) of oxCNDs@GPEI5K (**a**) and oxCNDs@GPEI25K (**b**) in D_2O containing maleic acid as internal standard.

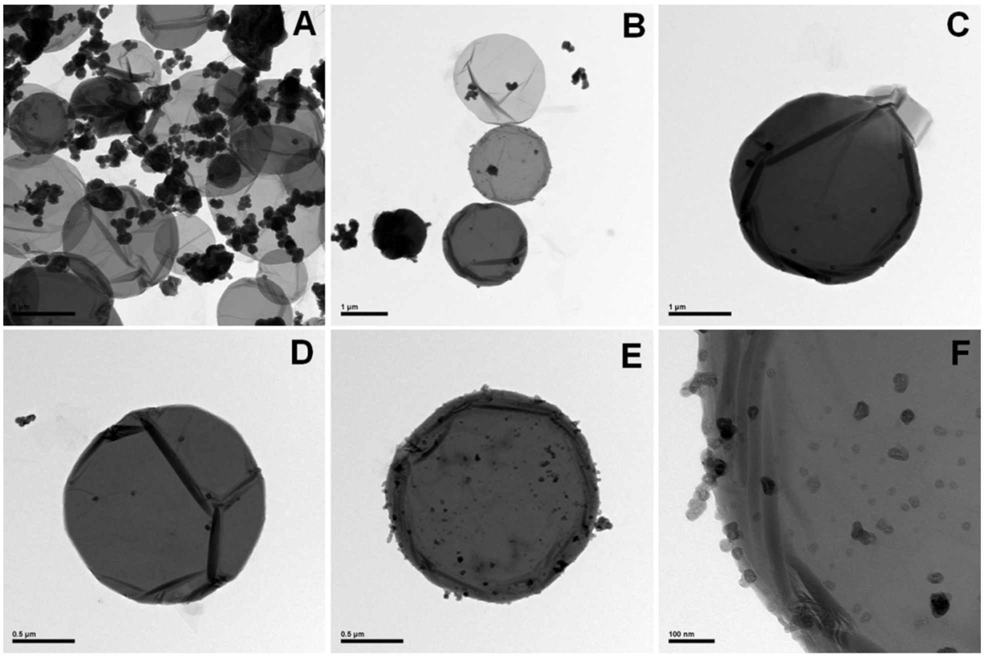

Figure S6. TEM images of oxCNDs.

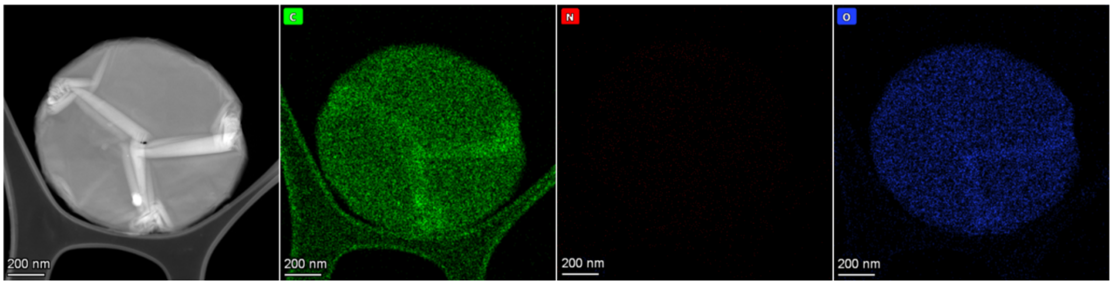

Figure S7. Scanning/Transmission Electron Microscopy high angle annular dark field images presenting the oxCNDs morphology, and the corresponding EDS elemental mapping images of C (K edge), N (K edge) and oxygen (K edge).

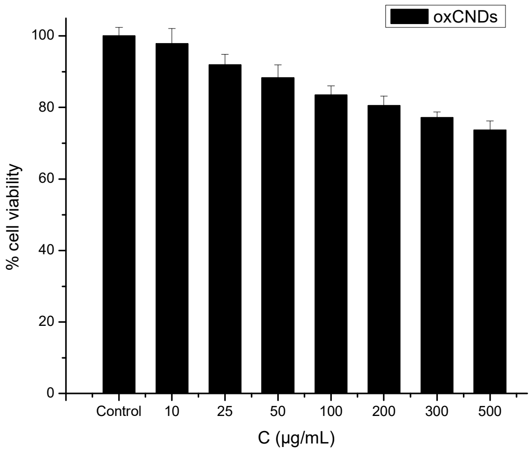

Figure S8. Comparative toxicities of the parent oxCNDs on human embryonic kidney HEK293 cells following incubation at various concentrations for 24 hours as determined by MTT assays. Data are expressed as mean ± SD of six independent values obtained from at least three independent experiments.

Table S1. Lorentzian peak fitting parameters for the first-order Raman bands of CNDs, oxCNDs, oxCNDs@GPEI5K, and oxCNDs@GPEI25K. $\Delta\tilde{\nu}$ = Raman shift/band position; FWHM = Full width at half maximum; *I* = Band intensity.

Sample	First-order Raman bands			
	**D**	**D″**	**G**	**D′**
**CNDs**	$\Delta\tilde{\nu}$ = 1355 cm^{-1}<br>FWHM = 34 cm^{-1}<br>*I* = 79	-	$\Delta\tilde{\nu}$ = 1583 cm^{-1}<br>FWHM = 21 cm^{-1}<br>*I* = 536	$\Delta\tilde{\nu}$ = 1623 cm^{-1}<br>FWHM = 33 cm^{-1}<br>*I* = 32
**oxCNDs**	$\Delta\tilde{\nu}$ = 1347 cm^{-1}<br>FWHM = 116 cm^{-1}<br>*I* = 4000	$\Delta\tilde{\nu}$ = 1499 cm^{-1}<br>FWHM = 129 cm^{-1}<br>*I* = 662	$\Delta\tilde{\nu}$ = 1571 cm^{-1}<br>FWHM = 61 cm^{-1}<br>*I* = 2561	$\Delta\tilde{\nu}$ = 1602 cm^{-1}<br>FWHM = 41 cm^{-1}<br>*I* = 2931
**oxCNDs@GPEI5K**	$\Delta\tilde{\nu}$ = 1347 cm^{-1}<br>FWHM = 112 cm^{-1}<br>*I* = 4288	$\Delta\tilde{\nu}$ = 1488 cm^{-1}<br>FWHM = 124 cm^{-1}<br>*I* = 611	$\Delta\tilde{\nu}$ = 1573 cm^{-1}<br>FWHM = 59 cm^{-1}<br>*I* = 3222	$\Delta\tilde{\nu}$ = 1606 cm^{-1}<br>FWHM = 36 cm^{-1}<br>*I* = 2618
**oxCNDs@GPEI25K**	$\Delta\tilde{\nu}$ = 1345 cm^{-1}<br>FWHM = 96 cm^{-1}<br>*I* = 3878	$\Delta\tilde{\nu}$ = 1468 cm^{-1}<br>FWHM = 179 cm^{-1}<br>*I* = 533	$\Delta\tilde{\nu}$ = 1574 cm^{-1}<br>FWHM = 67 cm^{-1}<br>*I* = 2701	$\Delta\tilde{\nu}$ = 1607 cm^{-1}<br>FWHM = 36 cm^{-1}<br>*I* = 2065

Table S2. Lorentzian peak fitting parameters for the second-order Raman bands of CNDs, oxCNDs, oxCNDs@GPEI5K, and oxCNDs@GPEI25K. $\Delta\tilde{\nu}$ = Raman shift/band position; FWHM = Full width at half maximum; *I* = Band intensity.

Sample	Second-order Raman bands		
	**G′ (2D)**	**D+G (D+D′)**	**2G (2D′)**
**CNDs**	$\Delta\tilde{\nu}$ = 2703 cm^{-1}<br>FWHM = 56 cm^{-1}<br>*I* = 551	-	-
**oxCNDs**	$\Delta\tilde{\nu}$ = 2706 cm^{-1}<br>FWHM = 432 cm^{-1}<br>*I* = 2385	$\Delta\tilde{\nu}$ = 2924 cm^{-1}<br>FWHM = 220 cm^{-1}<br>*I* = 1635	$\Delta\tilde{\nu}$ = 3172 cm^{-1}<br>FWHM = 553 cm^{-1}<br>*I* = 2420
**oxCNDs@GPEI5K**	$\Delta\tilde{\nu}$ = 2688 cm^{-1}<br>FWHM = 225 cm^{-1}<br>*I* = 1116	$\Delta\tilde{\nu}$ = 2923 cm^{-1}<br>FWHM = 281 cm^{-1}<br>*I* = 2123	$\Delta\tilde{\nu}$ = 3192 cm^{-1}<br>FWHM = 232 cm^{-1}<br>*I* = 1052
**oxCNDs@GPEI25K**	$\Delta\tilde{\nu}$ = 2686 cm^{-1}<br>FWHM = 266 cm^{-1}<br>*I* = 1072	$\Delta\tilde{\nu}$ = 2928 cm^{-1}<br>FWHM = 218 cm^{-1}<br>*I* = 1683	$\Delta\tilde{\nu}$ = 3181 cm^{-1}<br>FWHM = 163 cm^{-1}<br>*I* = 739

Table S3. Elemental analysis results of oxCNDs, GPEIs and GPEI-functionalized oxCNDs.

**Sample**	**Sample elemental composition/wt%**			**Polymer content (% w/w)**
	C	H	N	
**oxCNDs**	54.08	2.85	0.14	
**GPEI5K**	34.47	16.98	28.17	
**oxCNDs@GPEI5K**	52.49	2.43	3.96	13.63
**GPEI25K**	36.65	16.30	28.55	
**oxCNDs@GPEI25K**	52.58	3.09	4.76	16.26
